# Roxadustat: Do we know all the answers?

**DOI:** 10.17305/bb.2022.8437

**Published:** 2023-05-01

**Authors:** Qiu-Yu Li, Qian-Wen Xiong, Xuefeng Yao, Fei Liu, Xiaoxiao Tang, Haidong Fu, Tong Tong, Jianhua Mao, Wan-Xin Peng

**Affiliations:** 1 Department of Nephrology, The Children’s Hospital, Zhejiang University School of Medicine, National Clinical Research Center for Child Health, National Children’s Regional Medical Center, Hangzhou, China; 2 The Children’s Hospital, Zhejiang University School of Medicine, Hangzhou, China

**Keywords:** Erythropoietin (EPO), hypoxia-inducible factor prolyl hydroxylase inhibitor (HIF-PHI), roxadustat, renal anemia, therapeutic potential

## Abstract

Anemia is a common complication of chronic kidney disease (CKD), and its prevalence rises as the disease progresses. Intravenous or subcutaneous erythropoiesis-stimulating agents (ESAs) are advised to treat CKD-associated anemia, since shortage of erythropoietin (EPO) and iron is the main cause of anemia. However, ESA resistance and safety have spurred a lot of interest in the development of alternate anemia therapies. Roxadustat, an orally administered hypoxia-inducible factor prolyl hydroxylase inhibitor (HIF-PHI), which increases erythropoiesis and may modulate iron metabolism, was recently licensed in China, Chile, South Korea, Japan, and the European Union for the treatment of CKD-related anemia. Despite this, clinical trials have shown a number of adverse effects, including cardiovascular disease, hyperkalemia, and infections. Roxadustat’s potential effects on multiple organs and systems are also of concern. In this review, based on clinical evidence, we discuss the potentially detrimental effects of roxadustat to the known biology on systems other than kidney, and the need for long-term follow-up in order for roxadustat to be approved in more countries in the future.

## Introduction

Erythropoietin (EPO) deficiency is a predominant cause of anemia in chronic kidney disease (CKD) [1, 2], but anemia could be also caused by the complication of CKD. Thus, alleviating the burden of anemia is important to improve the quality of life and survival rate both in non-dialysis-dependent (NDD)-and dialysis-dependent (DD)-CKD patients [3]. Erythropoiesis-stimulating agents (ESAs) have been the standard of care for renal anemia and they have tremendously benefited patients by alleviating symptoms and eliminating the need for blood transfusions [4]. Despite its efficacy in treating anemia, adverse effects, including worsening hypertension, seizures, and dialysis access clotting, have been noted in patients receiving ESAs. Moreover, some patients with infections may be resistant to ESAs or need a higher ESAs dosage [5, 6]. Finally, due to inadequate gastrointestinal absorption of ESA, intravenous iron supplements are essential in end-stage renal disease (ESRD) patients. In particular, all of these treatments may increase the risk of infection, heart disease, and death in patients with kidney disease. Therefore, there is an urgent need for a better medication for anemia that is more potent and has fewer adverse effects than conventional ESAs [7].

**Figure 1.**
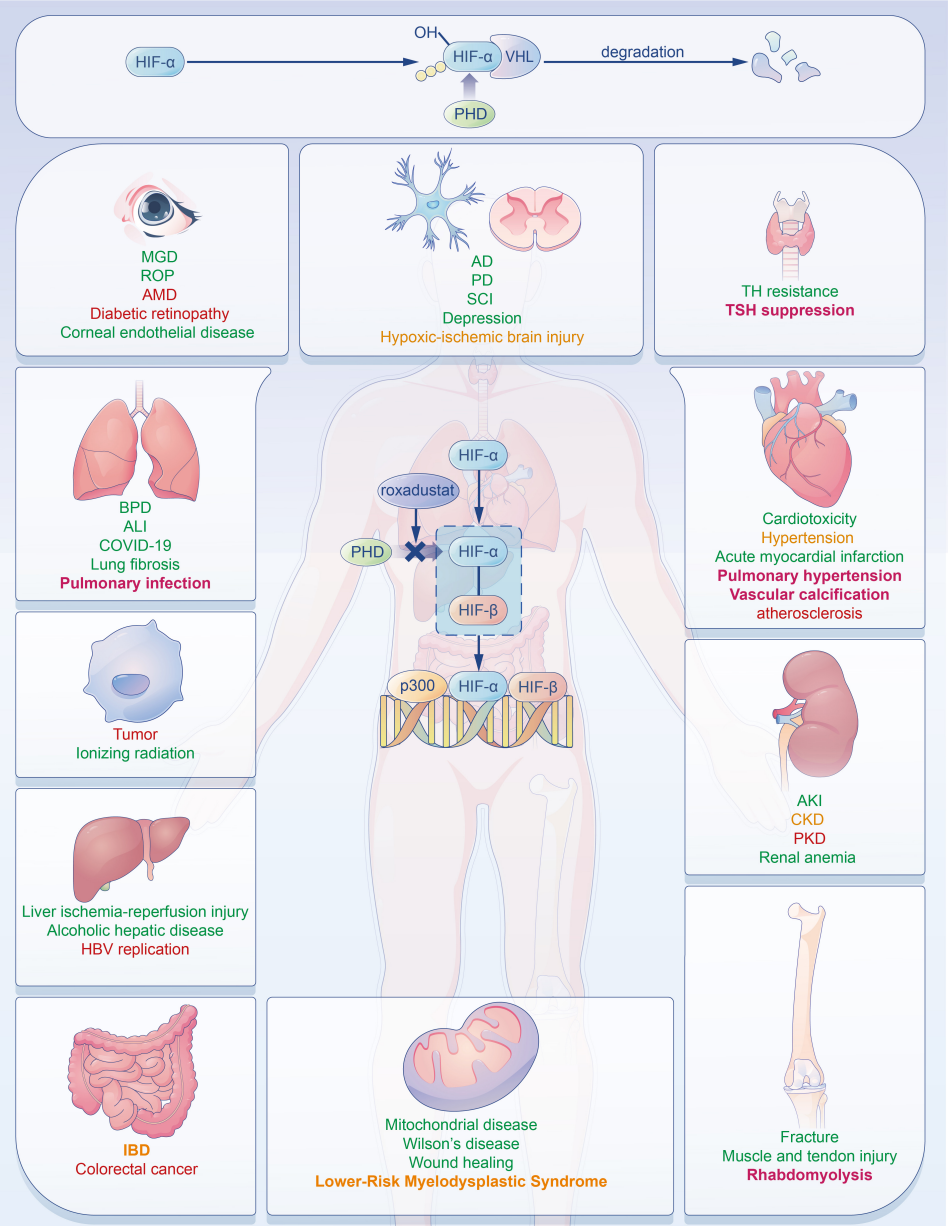
**The effects of roxadustat on multiple organs.** Roxadustat mediates the expression of many target genes and pathways and can exert its effects on multiple organs by inducing HIF stability. In this figure, diseases marked in green show potential positive effects of roxadustat, while those marked in red are potentially negative effects. These diseases marked in yellow are those whether roxadustat improves or worsens them are uncertain. Diseases highlighted in bold are clinically identified side effects of roxadustat to date. AKI: Acute kidney injury; CKD: Chronic kidney disease; PKD: Polycystic kidney disease; BPD: Bronchopulmonary dysplasia; ALI: Acute lung injury; AD: Alzheimer’s disease; PD: Parkinson’s disease; SCI: Spinal cord injury; MGD: Meibomian gland dysfunction; ROP: Retinopathy of prematurity; AMD: Age-related macular degeneration; TH resistance: Thyroid hormone resistance; TSH: Thyroid-stimulating hormone; IBD: Inflammatory bowel disease; HIF: Hypoxia-inducible factor.

The kidney, the main organ that senses changes in systemic oxygen tension, is the main source of EPO. EPO synthesis is regulated by hypoxia-inducible factor (HIF) in an oxygen-sensitive manner in the kidney [4, 8]. HIF is a heterodimer comprising two subunits, HIF-α and HIF-β. HIF-β is constitutively expressed, whereas HIF-α expression is oxygen concentration dependent. Under normoxic conditions, HIF-α is continuously produced and hydroxylated by prolyl hydroxylases (PHDs), which promotes the binding of von Hippel–Lindau protein (VHL) and leads to ubiquitination and proteasomal destruction of HIF-α. In conditions of low oxygen (hypoxia), the lack of O2 substrate inhibits PHD activity, resulting in the increase of HIF-α level [9, 10] (Figure 1, upper panel). The elevated HIF-α transcription activates the EPO gene. Circulating EPO then binds to the EPO receptor (EPOR) on red cell progenitors in the bone marrow, causing red cell mass to increase [11]. Due to the important role of HIF in EPO production, a strategy to manipulate the HIF pathway by using hypoxia-inducible factor prolyl hydroxylase inhibitor (HIF-PHI) has emerged as a novel approach for renal anemia management.

Roxadustat (FG-4592) is the first-in-class of small molecule HIF-PHI. By mimicking α-ketoglutarate, one of PHD’s substrates, Roxadustat, inhibits PHD and suppresses PHD’s role in regulating the balance between HIF synthesis and degradation rates, thereby correcting anemia. Roxadustat achieved its first approval in China for adults with DD-CKD in December 2018. Next year, it was approved for the treatment of renal anemia for NDD and DD patients in China. Now, roxadustat has also been approved in Japan, Chile, South Korea, and the European Union for the treatment of anemia in CKD in NDD and DD adult patients.

Because of the numerous downstream pathways involved, roxadustat may have multiple effects on various biological and physiological processes. Here, we review the function of roxadustat on multiple organs and some safety concerns it brings, chiefly focusing on long-term administration in patients with renal anemia and other renal diseases.

## Clinical effects of roxadustat

### Clinical effects on renal anemia

Roxadustat is confirmed to improve renal anemia through increasing EPO expression within a physiological range. It reduces elevated hepcidin levels in CKD patients. It also promotes iron release from intestinal cells into the blood and Fe2+ absorption, as well as improves iron transport with roxadustat’s target genes, divalent metal transporter-1 (DMT-1), duodenal cytochrome B (DCytB) [12]. A clinical trial (NCT02174627) was conducted on 2781 NDD-CKD patients who were followed up for four years. The results suggested that roxadustat could raise hemoglobin levels and decrease the rate of RBC transfusion significantly, even in 411 patients with elevated C-reactive protein (CRP). Subgroup analysis showed that serum iron and total iron-binding capacity levels increased, while ferritin and hepcidin levels decreased in the roxadustat group [13]. Similar results have also been obtained in clinical research conducted on NDD-CKD patients [13, 14].

Similar to the NDD-CKD clinical trial, hemoglobin response and the increase in the hemoglobin level was more efficient in the roxadustat group compared with the epoetin alfa group (88.4% vs 88.2%) among the 1043 incidences of dialysis for CKD patients. Hepcidin was always at a lower level in the roxadustat group compared with the epoetin alfa group [15]. Other clinical trials conducted on DD-CKD patients have also had similar outcomes [16, 17].

Anemia is also common in kidney transplant recipients. Early-onset anemia within six months post-transplantation mostly results from operative blood loss, repeated blood sample tests, and rejection, while late-onset anemia is mostly caused by chronic inflammation, allograft renal function reduction, and immunosuppressive therapies. Roxadustat treatment is also confirmed to have a therapeutic effect (improve Hb level and iron metabolism disorder) on post-transplant patients until treatment lasts for 2–4 weeks. Even though about 71.4% of patients are responsive, the responsive rate decreased in EPO-resistant patients and in patients with inflammation and impaired renal function [18, 19]. However, Naganuma et al. recommend a lower beginning dose for individuals with low body weight and iron supplementation, because iron delivery can produce a quick increase in Hb levels [20].

As reported, the dose of roxadustat was not associated with the hs-CRP level in patients with inflammation, while darbepoetin alfa was positively related [17]. Unfortunately, the number of cases analyzed is too small to provide meaningful statistical results, and thus, it failed to consider the change of CRP during the treatment. Therefore, a large number of cases are needed for renal anemia patients with high CRP levels.

### Clinical effects on cardiovascular diseases

The cardiovascular effects of roxadustat are of much attention. No significance of adverse events was found between roxadustat and placebo treatment. However, roxdustat seems not to decrease the incidence of cardiovascular diseases and fatal events compared to the ESA group. Serious adverse events are commonly seen in the roxadustat group vs the Epoetin Alfa group (14.2% vs 10%), among which cardiac and vascular disorders account for about 3.5% [13, 15, 16]. severe pulmonary arterial hypertension during roxadustat treatment is of great concern [21]. How to improve anemia but not increase cardiovascular risk is urgent. Hyperproliferation of pulmonary arterial smooth muscle cells (PASMCs), pulmonary vascular contraction caused by altered ion channel and ion transporter expression with HIF stabilization, and endothelial cell death dysregulated by VEGF and PGF lead to vascular occlusions and pulmonary hypertension [22]. During roxadustat treatment, vascular disease can be seen (1.0% vs 0.0%) [16]. The study focuses on vascular smooth muscle cell (VSMC) calcification, which is linked to vascular intimal calcification via increased pyruvate dehydrogenase kinase-4 (PDK4) and hexokinase-2 [23].

In addition, the incidence of arteriovenous fistula thrombosis seems to be greater in the roxadustat treatment group compared with the epoetin alfa group (9.0% vs 7.3%), even though roxadustat could not enhance platelet activation, production, and function in vivo and in vitro [15, 24, 25]. Several additional clinical studies have demonstrated that roxadustat can lower cholesterol levels through acetyl coenzyme A, which may contribute to the incidence of myocardial infarction [14, 26, 27]. Therefore, it is important to monitor and evaluate the risk of thrombosis and other cardiovascular events during clinical use.

### Clinical effects on other diseases

Based on the protective role exerted by roxadustat in anemia, roxadustat has been proven to be beneficial in anemia patients with lower-risk myelodysplastic syndrome who has low EPO level (<400 m IU/mL) and receive 1–4 RBC units infusion per eight weeks (NCT03263091). Transfusion independence (TI) was significantly effective in the roxadustat-treated group [28] in pure red cell aplasia (PRCA) patients who resist to EPO (rHu-EPO) treatment due to antibodies against EPO, can also be improved by roxadustat [29, 30].

Roxadustat can specifically combine with the hydrophobic space of thyroid hormone receptor-beta (THRβ) through the hydrophobic phenyl extension through a similar structure to T3, and activate the THRβ transcription, but without THRα overstimulation [31]. Roxadustat may be able to treat thyroid hormone (TH) resistance induced in part by THR gene mutations which is primarily in the ligand-binding domain of THR [32].

As reported, the integrity of the intestinal barrier may be mediated by HIF-1/2α stability with the barrier protective protein (CD73, intestinal trefoil factors) and anti-microbial protein expression [33]. However, it may promote inflammation and worsen dextran sulfate sodium (DSS)-induced colitis and intestinal colorectal cancer formation [34, 35]. The benefits of HIF-1α stabilizer on inflammatory bowel disease (IBD) also still need further studies (NCT04556383). In addition, roxadustat increases the incidence of infections [13, 17], potentially mediates cellular immunity, and inhibits humoral immunity [36, 37]. Finally, rhabdomyolysis was also observed three weeks after roxadustat treatment and re-administration in dialysis CKD patients [38].

Due to the multiple target genes and pathways of HIFs, it is understandable that roxadustat can have impacts on many other diseases. Thus, to better use of roxadustat in the clinic, it is important to acknowledge the potential effects of roxadustat on multiple systems and diseases.

## Potential effects of roxadustat

### Potential effects on acute/chronic kidney diseases

The pathophysiology of acute kidney injury (AKI) occurrence and AKI to CKD transition is a result of pyroptosis, ferroptosis, and necroptosis [39]. Pretreatment of roxadustat can prevent ischaemia–reperfusion (I/R) kidney injury through inhibiting inflammatory factors, attenuating mitochondrial injury, and decreasing Bax and cleaved caspase-3 levels and apoptosis [40]. Miao et al. demonstrated that roxadustat reduced I/R kidney injury by inhibiting the inflammatory response (lowering macrophages, neutrophils, and cytokines) and protecting tubular cells [41]. It can also downregulate nucleotide-binding oligomerization domain NOD-, LRR-, and pyrin domain-containing protein 3 (NLRP3) inflammasome activation, which is associated with pyroptosis in AKI [42]. Additionally, pretreatment with roxadustat impedes ferroptosis by reversing nuclear factor erythroid 2-related factor 2 (Nrf2) suppression caused by folic acid [43]. Roxadustat impedes AKI to CKD fibrotic transition by maintaining the redox balance and improving renal vascular regeneration associated with its target gene, EPO [44]. Otherwise, HIF stability can impede left ventricular hypertrophy and remodeling, which is caused by CKD. It ameliorates myocardial fibrosis through improving capillary density and mitochondrial morphology [45]. However, Kushida et al. [46] discovered that HIF-1 activates the transforming growth factor-β/sekelsky mothers against dpp3 (TGF-β/SMAD3) pathway and upregulates fibrosis-related genes. Besides anemia, the pro- or anti-fibrosis effect of roxadustat on CKD still needs to be confirmed (Figure 1).

Furthermore, HIF-1α stability is associated with the initiation and expansion of renal cyst through primary cilium loss and endothelial dysfunction in polycystic kidney disease (PKD) [47]. Therefore, it is necessary to closely monitor whether it leads to the formation of cysts in many organs and the enlargement of the original cysts [48]. HIF stability can also participate in activating prominin-1 positive stem cells and renal regeneration-specific proteins, such as nephrin and nestin in renal wound healing regeneration associated with decellularized (DC) renal scaffolds [48].

### Potential effects on cardiovascular diseases

It has been demonstrated that HIF-1α and the expression level of its target genes increased in heart samples undergoing acute ischemia and early infarction in 2000 [49]. Pre- and post-treatment with roxadustat enhanced the anaerobic process whereas HIF-1α inhibited aerobic respiration to produce more ATP under hypoxia and prevent cell death caused by hypoxia and myocardial infarction [50]. Additionally, it decreased the scar size, ventricular dilation, and improved cardiac repair and cardiac function via improving capillary density [51]. Vascular growth also contributes to cardiomyocyte repopulation [52]. HIF-1α, as the target of roxadustat, may contribute to the development of atherosclerosis in which inflammation and dyslipidemia are of the most importance. Lox-1 upregulation, endothelial and vascular inflammation caused by HIF-1α exacerbate foam cell formation and atherosclerosis [53]. VSMC proliferation regulated by HIF-1α secrets collagen and makeup fiber of plaque [54].

Previous studies have shown that the production of cardiac stem cells doubled at 5% O2 conditions, compared to 20% O2, with less senescent cells, better antioxidant stress resistance, and better recovery [55]. It is known that cardiac stem cells with overexpressing of HIF-1α can significantly promote angiogenic ability and blood flow recovery, as well as attenuate fibrosis in myocardial ischemia [56], suggesting that roxadustat may be valuable in cardiac stem cell therapy.

**Figure 2.**
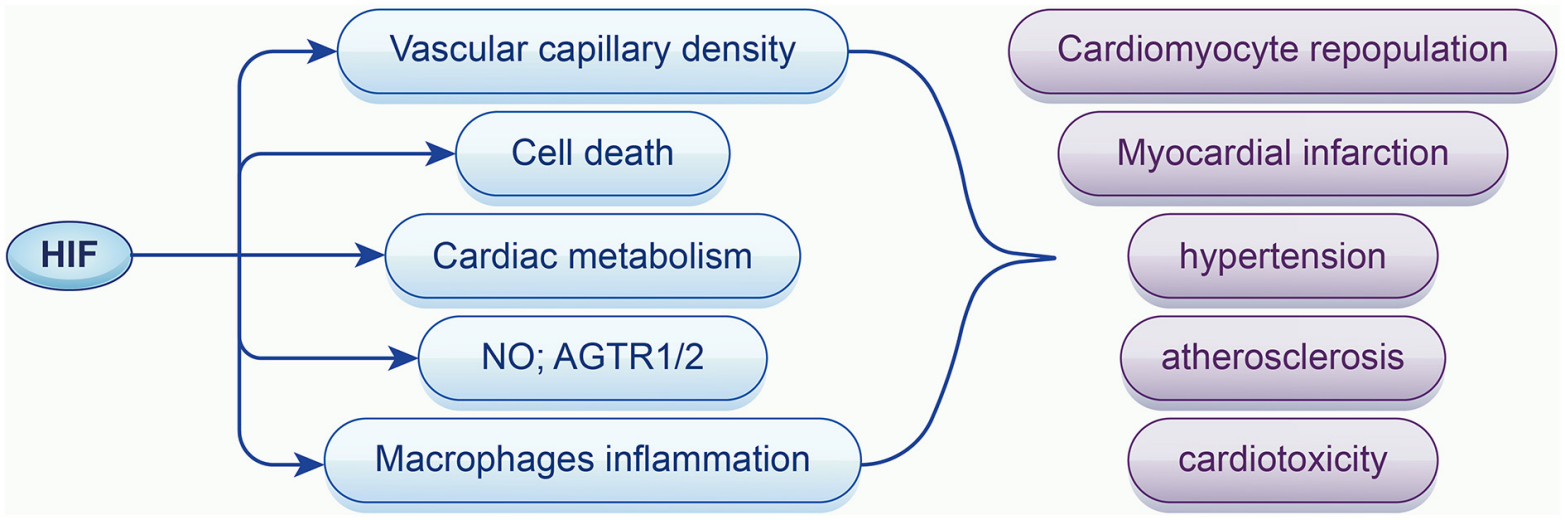
**The role of HIF on cardiovascular diseases.** HIF-1α stabilization regulates cardiovascular disease with target genes and pathways related to vascular capillary density, cell apoptosis, cardiac metabolism, NO (nitric oxide), ROS (reactive oxygen species), and inflammation; HIF: Hypoxia-inducible factor.

In the vascular system, roxadustat seems to dilate vascular smooth muscles and alleviates hypertension induced by Ang-II via promoting the release of NO and angiotensin receptor type 1 (AGTR1/2) expression with HIF-1α accumulation [57]. In contrast, HIF-2α is supposed to decrease the production of NO, which may produce opposite effects in hypertension [58]. Moreover, roxadustat attenuates cardiotoxicity induced by doxorubicin by inhibiting apoptosis and reactive oxygen species (ROS) production (Figure 2) [59].

### Potential effects on respiratory diseases

The lung is an organ that exchanges oxygen and carbon dioxide. Both hyperoxia and hypoxia may result in lung injury. In preterm, roxadustat can prevent bronchopulmonary dysplasia (BPD) by promoting pulmonary angiogenesis and pulmonary alveolarization [60]. Compared with vadadustat, roxadustat could significantly increase the pulmonary volume and alveolar volume, as well as improve exercise tolerance and compensatory lung growth [61]. In addition to lung development, roxadustat can relieve airway inflammation and protect against acute lung injury (ALI) by increasing heme oxygenase-1 (HO-1) expression and decreasing tumor necrosis factor-α and interleukin-1β production [62]. However, HIF-1α is abundant in chronic obstructive pulmonary disease, which may interact with cytokines through epidermal growth factor receptor (EGFR)/phosphoinositide 3-kinase (PI3K)/protein kinase B (AKT) pathway and further up-regulate HIF-1α self [63]. Given the protective effect exerted by roxadustat on renal tubulointerstitial inflammation, Huang et al. [64] proposed that roxadustat could reduce alpha-smooth muscle actin (α-SMA), connective tissue growth factor, and collagen I/III expression to mitigate the proliferation of lung fibroblasts. Roxadustat can reduce the pathology score and ameliorate pulmonary fibrosis, although it may be impossible to recover to initial levels. Roxadustat may be beneficial to COVID-19 patients as it can reduce the level of angiotensin-converting enzyme-2 (ACE2) and transmembrane protease serine 2 (TMPRSS2), which is associated with the entrance and replication of SARS-CoV-2 in lung epithelial cells [65]. Furthermore, EPO, as a target of roxadustat, which is known as an anti-inflammatory cytokine and prevents apoptosis, is now able to relieve severe COVID-19 [65].

### Potential effects on neurological diseases

Alzheimer’s disease (AD) accounts for the majority of neurodegenerative diseases. It is characterized by amyloid beta-peptide accumulation in the brain and neurofibrillary tangles composed of hyperphosphorylated protein with an HIF decrease, which can be reversed by roxadustat [66]. However, further research is still needed to confirm the finding suggested by Mitroshina that HIF-1α may increase the risk of AD through the increase in amyloid-beta precursor protein processing [67]. Parkinson’s disease (PD), which is secondary only to AD, is characterized by the loss of neurons and Lewy bodies along with the presentation of α-synuclein in the substantia nigra caused by mitochondrial dysfunction, oxidative stress, and apoptosis. Roxadustat prevents 1-methyl-4-phenyl-pyridiniumion induced by PD by promoting mitochondrial respiration and autophagy, mitigating oxidative stimulation and the inflammatory response, and finally by protecting nigral dopaminergic cells. It also enhances neuroblastoma (SH-SY5Y) cellular viability and reduces apoptosis along with HIF stability [68].

In contrast to other organs, the effect of Roxadustat on hypoxic-ischemic brain injury remains unknown. HIF-1 reduces hypoxia-induced brain damage when administered prior to ischemia or after four days of hypoxia (>4 days), but it may promote cell death within 24 h of ischemia [69]. The effect of HIF-1α on apoptosis relies on the severity of hypoxia. Under mild hypoxia, HIF-1 protects cells via anti-apoptotic factors, whereas in severe hypoxia, it causes cell death via the pro-apoptotic protein p53 [67]. Roxadustat may also be associated with an increase in the permeability of the blood–brain barrier caused by the HIF-1α/VEGF pathway [70], which may be associated with intracranial infection. As a result, it is critical to confirm roxadustat’s function in hypoxic-ischemic brain damage and to determine the best time to use roxadustat.

Traumatic spinal cord injury (SCI) is a devastating disease with no specific effective treatments. Roxadustat treatment inhibits tert-butyl hydroperoxide-induced apoptosis, increases nerve cell survival, and promotes neuronal repair in SCI recovery [71]. Roxadustat also contributes to decreasing the apoptosis of stem cells and to improve the tolerance and survival rate of stem cells after transplantation [72].

Roxadustat is also involved in the treatment of depression, which is caused by neurological dysfunction. Roxadustat improves memory impairment by reversing the decline in memory-associated cAMP response element-binding protein (CREB)/brain-derived neurotrophic factor (BDNF) signals in the hippocampus. Similarly, the reduction in synaptic density proteins caused by chronic unpredictable mild stress can also be reversed by roxadustat [73]. Synaptic growth and neurogenesis are both effective strategies. Additionally, EPO also contributes to antidepressant effects by promoting the expression of neurotrophic genes, such as BDNF and a neuropeptide precursor VGF [74].

### Potential effects on ophthalmic diseases

Roxadustat is also useful in the treatment of many ophthalmic diseases. Meibomian gland dysfunction (MGD) is the major reason for dry eye disease and has no specific treatment. Roxadustat may be a novel method of treatment for MGD because it facilitates immortalized human meibomian gland epithelial cell differentiation and causes acidic conditions that activate DNase II, which is linked to programmed cell death and holocrine secretion. HIF can also boost the content of microbodies and neutral lipids, which eventually improve MGD [75]. Compared with the effect of dimethyloxallyl glycine (DMOG), which mainly exerts its effects on the liver, intermittent administration of roxadustat prevents oxygen-induced retinopathy of prematurity (ROP) in two main ways. It directly induces normal sequential retinal neovascular growth and reduces the extent of the vascular loss matrix. On the other hand, roxadustat promotes angiogenesis by increasing the expression of serum angiokines via hepatic HIF-1α stabilization [76].

Endothelial keratoplasty is the main type of optical surgery conducted for a corneal endothelial disease that can aid in the recovery of the wound and restore vision quickly, but the pressure exerted during surgery may lead to the loss of corneal cells from the donor endothelium. Pretreatment with roxadustat can effectively prevent mechanical stress induced by sonication and diminish endothelial cell loss by up to 23%, protecting endothelial cells from oxidative stress and apoptosis in vitro [77]. HIF-1α stabilization by roxadustat can also improve mitophagy and protect against photoreceptor injury caused by experimental retinal detachment [78]. However, with its target gene, VEGF, roxadustat may cause leaky blood vessels edema stemming and the development of retinal lesions and apoptosis, particularly in dialysis patients with diabetic retinopathy and age-related macular degeneration (AMD) [79].

### Potential effects on hereditary metabolic diseases

Mitochondrial respiratory chain monogenic disease, which is characterized by oxygen poisoning caused by impaired oxygen utilization, continuous oxygen transport, and utilization mismatch, is rare but is the largest category of congenital metabolic disorders without an effective method of treatment. Jain et al. [80] found that the exogenous addition of roxadustat could effectively prevent the mitochondrial respiratory chain from damage and completely rescue growth defects by triggering the extensive hypoxia transcription program, increasing glycolysis of HEK293T cells by up to 25%, and reducing the production of ROS.

In Wilson’s disease, roxadustat can decrease the level of hepatic Cu, improve hepatic steatosis and neurological symptoms by upregulating the expression levels of copper transporters, as well as improving hepatic metabolism by decreasing the expression of lipogenic genes [81, 82].

Roxadustat may be effective for diabetes and diabetic nephropathy through its target genes, glucose transporter (GLUT), and EPO. GLUT-1 improves the absorption of glucose, while GLUT4 reinforces the sensitivity of insulin. Moreover, EPO is involved in restraining inflammation and gluconeogenesis. HIF-2α stability decreases postprandial glucagon signaling and gluconeogenesis through ERK 1/2-dependent-phosphodiesterases [83].

### Potential effects on cancer

The small lumen of tumor vasculature and poor tight junctions result in hypoxia of the tumor microenvironment, which is positively associated with tumor progression and metastasis associated with high interstitial pressure. Roxadustat can normalize tumor microenvironment (TME) and restore tumor blood supply, which may restrain the progression and metastasis of the tumor. Furthermore, roxadustat can directly regulate Ly6Clo macrophages and transform them into the phagocytic phenotype, which can improve the tumor vessel lumen area and activate the phagocytic ability for tumor defense [84]. Even though Seeley et al. [85] believed that roxadustat treatment had no influence on tumor initiation, progression, and metastasis in a breast cancer mouse model, some researchers have speculated that roxadustat has an effect on tumor tumorigenesis and progression but this effect is still unclear. HIF-1α may contribute to tumor onset, while HIF-2α can mediate tumor formation and growth [86]. Therefore, it is essential to monitor tumor risk factors during the treatment of roxadustat.

Except for the effect on the tumor itself, roxadustat can enhance the antitumor effects of doxorubicin [59]. Roxadustat also plays a protective role in ionizing radiation. Pretreatment with roxadustat can protect the hematopoietic system from radiation-induced bone marrow structure damage, restoring the number of nucleated cells and hematopoietic function of the bone marrow [87]. Similarly, gastrointestinal radiation injury is also prevented with roxadustat pretreatment by decreasing the apoptosis of epithelial cells and promoting intestinal stem cell regeneration through Wnt/β-catenin pathway activation [88].

### Potential effects on other diseases

Roxadustat therapy reduces liver I/R injury through improving hepatic cell ballooning and steatosis while also promoting revascularization stability [89, 90]. It has also been reported to protect against alcoholic hepatic disease by suppressing inflammation and ROS [91, 92]. Hepatic HIF-1α stabilization, on the other hand, is likely to be associated with the development and poor prognosis of hepatocellular carcinoma and hepatic fibrosis, as well as promote HBV replication through activating basic core promoter [93, 94].

Roxadustat administration promotes mature osteoclast bone resorption and new bone formation following a fracture, as well as bone marrow stem cells (BMSCs) proliferation and migration to fracture sites [95]. Otherwise, as target genes of roxadustat, EPO and VEGF promote muscle angiogenesis and muscle repair caused by trauma or physical exercise [95]. Roxadustat promotes wound healing by boosting neovascular angiogenesis and improving the proliferation and movement of epidermal stem cells [96].

## Conclusion

Orally accessible roxadustat appears to open a new door for the treatment of renal anemia in patients with acute or chronic inflammation, even in a dose-dependent manner, based on its positive effects on renal anemia. In addition to its more convenient form, roxadustat is more cost-effective than placebo [97]. Compared to ESA, roxadustat looks to be more cost-effective due to its lower cost and the higher quality of life for patients.

Although roxadustat’s effects on renal anemia are well-established, its effects on post-transplant anemia remain restricted. There have been very few trials [18, 19]. While these studies demonstrate the efficacy of roxadustat in treating post-transplant anemia, further clinical study is required. Systemic or localized inflammation may be associated with EPO resistance [98].

Some clinical trials suggest that roxadustat is beneficial for treating renal anemia in people with inflammation, however, the sample sizes are insufficient to give conclusive evidence. In addition, larger clinical trials must concentrate on the initial intensity of inflammation and the evolution of inflammation following treatment. Overall, we anticipate more applications for roxadustat in anemia with inflammation, EPO-resistant anemia, and post-transplant anemia.

Every coin has two sides to it. Roxadustat’s multi-targeting properties raise some safety concerns. The most common issue is cardiovascular risk, which is related to the rate of Hb improvement and the highest level of Hb. It is critical to understand ways to reduce cardiovascular risk. Although standard-dose treatment may be more successful in increasing Hb, it may be associated with a higher risk of unexpected adverse effects [99]. A lesser dose of roxadustat can also attain the optical Hb level, which gradually improves Hb [100], although patients who use a lower dose may have a better anemic state. Choosing a varied dose for patients with varying hemoglobin levels may help to reduce the occurrence of cardiovascular events. Another concern is the duration of roxadustat medication. Although short-term treatment has good effects, long-term therapy may have some additional detrimental effects. Long-term, large-scale clinical trials focusing on varied doses for individuals with varying illness severity are desperately needed.

In addition to the recognized effects, hypothetical impacts are combined. Exogenous transplantation of HIF-PHIs may upset the intrinsic equilibrium, leading to the disruption of other pathways and the dysfunction of other organs, including the exacerbation of diabetic retinopathy, the growth of malignancies, and the enlargement of cysts. Candidates for roxadustat treatment should be concerned. Targeted therapy may provide a solution. However, we are still cautious when diabetic nephropathy is the underlying cause of renal anemia. In addition, tests for heart illness, cyst disease, and malignancies are still necessary even when focused therapy is utilized in the clinic.

Additionally, Daprodustat, Vadadustat, Enarodustat, and Molidustat are the subject of extensive preclinical and clinical studies. Different drugs inhibit certain PHDs and produce distinct effects. For instance, Daprodustat, which inhibits PHD1 and PHD3 preferentially, demonstrated a risk of cancer in addition to cardiovascular problems. Hypertension is comparatively common in vadadustat. Although several prior publications have outlined the roles of HIF-PHIs in diverse systems [101], this article provides a comprehensive overview. In the present review, we concentrate on roxadustat. Notably, we describe not only its major benefits in ameliorating renal anemia and its prospective therapeutic applications in other diseases but also other safety concerns that may occur during treatment. We hope that our analysis will be helpful when roxadustat is being evaluated for the treatment of a condition. Particular care should be paid to the time, patient selection, and dosage of roxadustat in order to expand its global utilization.
